# Geographic variation in sexual behavior can explain geospatial heterogeneity in the severity of the HIV epidemic in Malawi

**DOI:** 10.1186/s12916-018-1006-x

**Published:** 2018-02-09

**Authors:** Laurence Palk, Sally Blower

**Affiliations:** Center for Biomedical Modeling, Semel Institute of Neuroscience and Human Behavior, David Geffen School of Medicine, University of California, 760 Westwood Plaza, Office 27-423, Los Angeles, California 90095 USA

**Keywords:** HIV, Sexual behavior, Epidemiology, Malawi, Sub-Saharan Africa, Geostatistics

## Abstract

**Background:**

In sub-Saharan Africa, where ~ 25 million individuals are infected with HIV and transmission is predominantly heterosexual, there is substantial geographic variation in the severity of epidemics. This variation has yet to be explained. Here, we propose that it is due to geographic variation in the size of the high-risk group (HRG): the group with a high number of sex partners. We test our hypothesis by conducting a geospatial analysis of data from Malawi, where ~ 13% of women and ~ 8% of men are infected with HIV.

**Methods:**

We used georeferenced HIV testing and behavioral data from ~ 14,000 participants of a nationally representative population-level survey: the 2010 Malawi Demographic and Health Survey (MDHS). We constructed gender-stratified epidemic surface prevalence (ESP) maps by spatially smoothing and interpolating the HIV testing data. We used the behavioral data to construct gender-stratified risk maps that reveal geographic variation in the size of the HRG. We tested our hypothesis by fitting gender-stratified spatial error regression (SER) models to the MDHS data.

**Results:**

The ESP maps show considerable geographic variation in prevalence: 1–29% (women), 1–20% (men). Risk maps reveal substantial geographic variation in the size of the HRG: 0–40% (women), 16–58% (men). Prevalence and the size of the HRG are highest in urban centers. However, the majority of HIV-infected individuals (~75% of women, ~ 80% of men) live in rural areas, as does most of the HRG (~ 80% of women, ~ 85% of men). We identify a significant (*P* < 0.001) geospatial relationship linking the size of the HRG with prevalence: the greater the size, the higher the prevalence. SER models show HIV prevalence in women is expected to exceed the national average in districts where > 20% of women are in the HRG. Most importantly, the SER models show that geographic variation in the size of the HRG can explain a substantial proportion (73% for women, 67% for men) of the geographic variation in epidemic severity.

**Conclusions:**

Taken together, our results provide substantial support for our hypothesis. They provide a potential mechanistic explanation for the geographic variation in the severity of the HIV epidemic in Malawi and, potentially, in other countries in sub-Saharan Africa.

## Background

Substantial geographic variation in the severity of epidemics has been observed for many infectious diseases, e.g., malaria, onchocerciasis, and schistosomiasis [1–4]. This variation has been shown to be the result of geographical variation in conditions that affect transmission. Notably, there is substantial geographic variation in the severity of HIV epidemics [5–7] in sub-Saharan Africa, but the underlying mechanistic determinants of this variation have not been identified. In sub-Saharan Africa, ~ 25 million individuals are infected with HIV [8], transmission is predominantly heterosexual, and prevalence is high in the general population. An individual’s most important risk factor for acquiring HIV is their number of sex partners: the greater the number of sex partners, the greater the risk. We hypothesize that geographic variation in the size of the high-risk group (HRG) generates geographic variation in the severity of HIV epidemics in sub-Saharan Africa; the HRG is defined as the group of individuals who have a high number of lifetime sex partners. We test our hypothesis by conducting a spatial analysis of georeferenced HIV testing and sexual behavior data collected from ~ 14,000 individuals during a nationally representative population-level survey in Malawi: the 2010 Malawi Demographic and Health Survey (MDHS) [9].

**Fig. 1:**
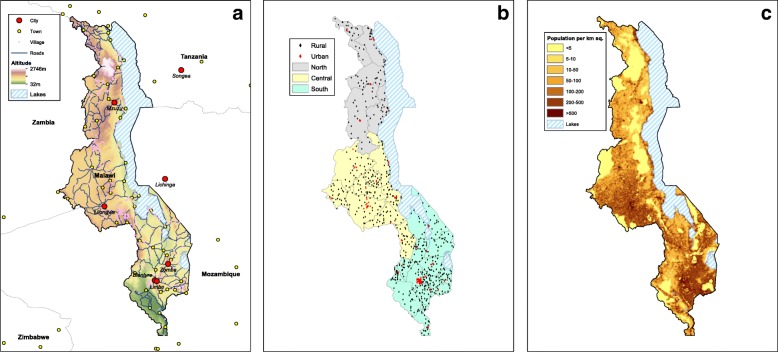
**a** Map of Malawi. Background colors reflect topography; the altitude scale is in meters. *Blue lines* show roads. Cities and towns in Malawi and surrounding countries are shown in *red* and *yellow*, respectively. Villages in Malawi are shown in *pink*, and *blue-striped* areas represent lakes. **b** Map showing the sample cluster locations for the Demographic and Health Survey conducted in 2010 in Malawi. Rural locations are shown by *black diamonds*, urban locations by *red diamonds*. The three administrative regions in Malawi are color-coded: North (*gray*), Central (*yellow*), and South (*blue*). *Black lines* mark the boundaries of the 27 districts that were included in the survey. **c** The population density map for Malawi. The color code shows the number of individuals per square kilometer

HIV prevalence in Malawi is high; nationwide, ~ 13% of women and ~ 8% of men are infected with the virus [9]. Malawi is divided into three administrative regions: Northern, Central, and Southern (Fig. 1a). Communities are predominantly urban in the Southern region, semi-urban in the Central region, and rural in the Northern region. There is a major urban center in each region: Mzuzu in the Northern region, Lilongwe (the capital) in the Central region, and Blantyre and Zomba in the Southern region (Fig. 1a). Lilongwe is the largest city in Malawi, Blantyre the second largest, and Mzuzu the third. The majority of the population of ~ 15 million individuals live in the Central and Southern regions: 42% and 45%, respectively. Notably, 85% of the population lives in rural communities [10].

Previous studies have focused on developing complex statistical models to predict the prevalence of HIV [6, 11–13]. These models include multiple risk factors (e.g., circumcision and condom usage) and non-causal descriptive determinants, e.g., distance to a road. They have shown that there can be considerable small-scale heterogeneity in HIV prevalence and in risk behaviors. However, none of these models tested the hypothesis that we are proposing, nor have they identified a mechanism that explains a significant proportion of the geographic variation in the severity of HIV epidemics in sub-Saharan Africa.

We conducted our analyses in three stages. First, we used data from the MDHS to quantify the geographic variation in the severity of the HIV epidemic in Malawi. Specifically, we constructed gender-stratified maps of prevalence, where prevalence is the proportion of the population that is infected with HIV. Second, we used data from the MDHS to construct gender-stratified risk maps that show the geographic variation in the size of the HRG. Finally, we tested our hypothesis by using the MDHS data to construct gender-stratified spatial error regression (SER) models. Data were analyzed at the level of the district; Malawi is divided into 28 administrative districts (Fig. 1a). The district of Likoma, which consists of two islands in Lake Malawi, was not included in the MDHS. Therefore, our analysis is based on data from 27 districts. The statistical models that we constructed enabled us to identify a geospatial relationship that quantifies the effect of the size of the HRG on increasing (and decreasing) HIV prevalence.

## Methods

### Demographic mapping

We used data from the WorldPop database to construct a demographic map of Malawi [14]. The resultant map shows the estimated number of individuals in each square kilometer in Malawi.

### HIV testing and behavioral data

We used georeferenced data from the 2010 MDHS [9]; these data are publicly available. The survey sample sites are distributed, in proportion to the population, among 27 districts: red diamonds show urban sites, black show rural (Fig. 1b). The response rate to the survey was very high: 97% for women, 92% for men. Ninety-one percent of eligible women and 84% of eligible men were tested for HIV. Each individual’s HIV test results were linked to that person’s demographic and behavioral data. We used data from the 7396 women and 6509 men, aged 15–49 years old, who were tested for HIV. Since response rates were so high, we did not need to adjust the HIV prevalence results for non-participation [15].

### Risk groups and HIV acquisition

We used MDHS data to calculate, for men and women, the percentage of individuals who acquired the virus through low-risk and high-risk behavior. For example, we calculated the fraction of HIV-infected women in the high-risk group, *F*, as follows:

1 $$ F=\frac{Nhq}{Np}=\frac{hq}{p} $$

where *N* is the number of women aged 15–49, *p* is the national prevalence of HIV in women, *h* is the fraction of women who are in the HRG, and *q* is the prevalence of HIV in the HRG of women.

We made similar calculations for low-risk women and for men.

### Gender-stratified epidemic surface prevalence maps

To construct the maps, we spatially smoothed and interpolated the georeferenced HIV testing data from the MDHS. The epidemic surface prevalence (ESP) maps show the percentage of individuals (15–49 year olds) who are infected with HIV. We used an adaptive bandwidth kernel density estimation method, with a two-dimensional Gaussian for the kernel density function, to construct the maps [16]. We chose a ring size of 200 individuals for smoothing; this ensured the smoothing circle included a minimum of 200 individuals and at least three other sampling sites. The R programming package prevR was used for implementation [17].

### Gender-stratified risk maps

To construct these maps, we used the 2010 MDHS data and the same mapping techniques that we used to construct the ESP maps. Each risk map shows the percentage of 15–49 year olds who have had a high number of sex partners over their lifetime, i.e., the size of the HRG. We used the lifetime number of sex partners as it represents the cumulative risk of acquiring HIV.

### Gender-stratified regression models

We tested our hypothesis by fitting regression models to the MDHS data; the data were stratified by gender and district. HIV prevalence was the response variable, and the size of the HRG was the explanatory variable. We used both a SER model (Eq. 2) and an ordinary least squares regression model (Eq. 3). The SER model accounts for the fact that error terms that are geographically close are more likely to be similar and therefore spatially auto-correlated [18]. We compared the fit of the regression models to determine if the SER models were the most appropriate for our analysis.

2 $$ p= X\beta +\gamma \kern0.5em \mathrm{where}\kern0.5em \gamma =\lambda {W}_{\gamma }+u $$

3 $$ p= X\beta +\varepsilon $$

In both equations, *p* represents a vector of the prevalence of HIV in each district, *X* a vector of the size of the HRG in each district, and *β* is the regression coefficient. In the SER model (Eq. 2), *γ* is the spatially auto-correlated error, *λ* is the auto-regressive coefficient, *W*_*γ*_ is the spatial error lag term, and *u* specifies the non-spatial random error with mean zero. We used Queen’s contiguity [19] to assign spatial weights for districts; i.e., we assigned non-zero weights to neighboring districts that share a common edge or vertex, and zero weights to the other districts. Different weighting methods could be applied; we used Queen’s contiguity, as it was the most parsimonious. In the ordinary least squares regression model (Eq. 3), *ε* specifies the random error with mean zero.

We used the gender-stratified regression models to quantify the effect of the size of the HRG on increasing (and decreasing) HIV prevalence. We estimated the percentage of the geographic variation in HIV prevalence that could be explained by the geographic variation in the size of the HRG.

### Residual maps

To show the goodness of fit of the gender-stratified spatial regression models, we mapped the residual values. These maps show where the models under/overestimate prevalence, and therefore they indicate the areas where there may be confounders.

## Results

The demographic map reveals the geospatial distribution of all individuals living in Malawi (Fig. 1c). The map shows the size of settlements, the settlement dispersion patterns, and geographic variation in population density. There are clear differences in the spatial demographics of the three regions. Communities are comparatively large and close together in the Southern region, tend to be smaller and more dispersed in the Central region, and are fairly small and widely dispersed in the Northern region. Substantial urban-rural differences are apparent. The population density ranges from less than five individuals per square kilometer in rural areas to more than 500 individuals per square kilometer in the major urban centers: Lilongwe, Blantyre, Zomba, and Mzuzu (Fig. 1c).

**Fig. 2:**
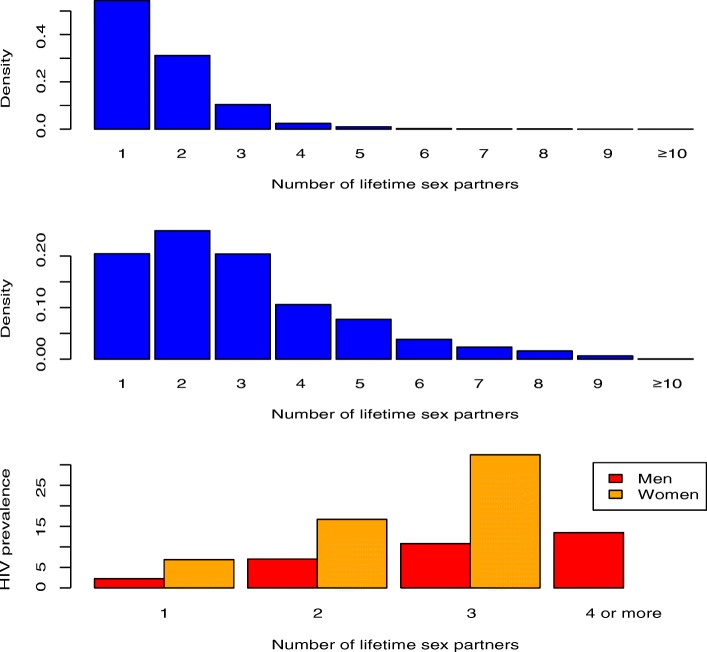
**a** Histogram showing the distribution of the number of lifetime sex partners for women (aged 15–49 years old). Data are from the 7396 women in the 2010 Malawi Demographic and Health Survey (MDHS) who were tested for HIV. **b** Histogram showing the distribution of the number of lifetime sex partners for men (aged 15–49 years old). Data are from the 6509 men in the 2010 MDHS who were tested for HIV. **c** HIV prevalence (%) stratified by number of lifetime sex partners. Data are from the 7396 women and 6509 men in the 2010 MDHS who were tested for HIV

The distribution of sexual behaviors in the general population in Malawi is shown for women (Fig. 2a) and men (Fig. 2b). Most women (~ 85%) have only had one or two lifetime sex partners; however, their risk of acquiring HIV has been substantial, 7% and 17%, respectively (Fig. 2c). We define the HRG of women as those who have had three or more lifetime partners; almost a third of these women have become infected with HIV (Fig. 2c). In comparison with women, as with all sexually transmitted diseases, men have had a lower risk of acquiring HIV (Fig. 2c). We refer to the group of men who have had four or more lifetime sex partners as the HRG; ~ 14% of men in the HRG have acquired HIV (Fig. 2c). Notably, we find that ~ 66% of HIV-infected women and ~ 50% of HIV-infected men in Malawi acquired the virus even though they were not in the HRGs; i.e., they acquired HIV through “low-risk” behavior.

**Fig. 3:**
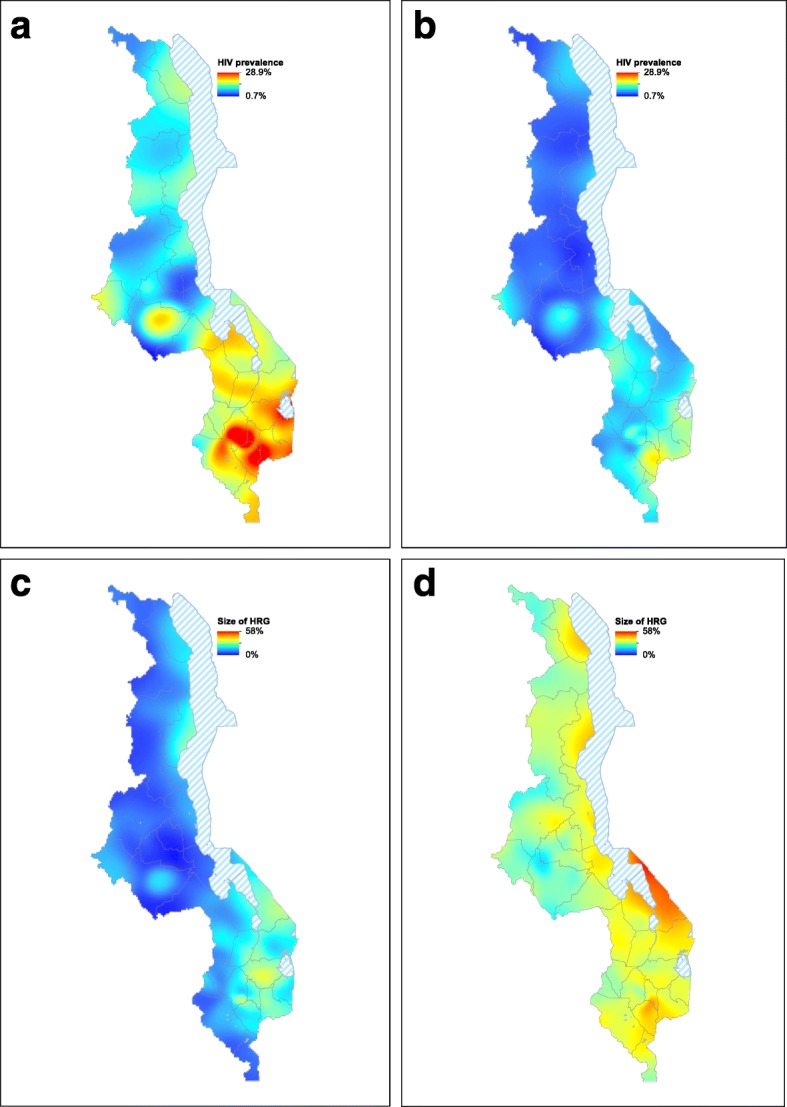
**a** HIV epidemic surface prevalence (ESP) map for women (15–49 years old). Prevalence is shown as the percentage of women who are infected with HIV. Data used to construct the map are from the 7396 women in the 2010 Malawi Demographic and Health Survey (MDHS) who were tested for HIV. **b** HIV ESP map for men (15–49 years old). Prevalence is shown as the percentage of men who are infected with HIV. Data used to construct the map are from the 6509 men in the 2010 MDHS who were tested for HIV. **c** Map showing, for women, geographic variation in the size of the HRG. The size of the HRG is defined as the percentage of women (15–49 years old) who have had three or more lifetime sex partners. Data used to construct the map are from the 7396 women who participated in the 2010 MDHS and were tested for HIV. **d** Map showing, for men, geographic variation in the size of the HRG. The size of the HRG is defined as the percentage of men (15–49 years old) who have had four or more lifetime sex partners. Data used to construct the map are from the 6509 men who participated in the 2010 MDHS and were tested for HIV

The ESP maps provide a spatial visualization of the severity of the HIV epidemic in Malawi: Fig. 3a (women), Fig. 3b (men). Overall, prevalence is ~ 13% in women, ~ 8% in men. However, there is considerable geographic variation in severity; prevalence varies from 1 to 29% in women and from 1 to 20% in men. Prevalence is almost always higher in women than in men. Large-scale spatial patterns are apparent, and they are more distinct for women than for men. There is a strong North-South trend in increasing prevalence, and a sharp contrast between urban and rural areas in all three regions. Prevalence is highest in Blantyre and Zomba in the South, and Lilongwe in the Central region. Prevalence is also fairly high in fishing villages along Lake Malawi and around Mzuzu in the North. Notably, although prevalence is higher in urban centers than in rural areas, ~ 75% of Malawi’s HIV-infected women and ~ 80% of HIV-infected men live in rural communities. This is due to the fact that most of Malawi’s population lives in rural areas.

The gender-stratified risk maps show that there is considerable geographic variation, among communities, in the size of the HRG: Fig. 3c (women), Fig. 3d (men). This geographic variation results in large-scale spatial patterns that appear similar to the patterns in the ESP maps. The size of the HRG of women — in a community — varies from 0 to 40%, whereas the size of the HRG of men — in a community — varies from 16 to 58%. In almost all communities, the size of the HRG of men is greater than the size of the HRG of women. There are discernable large-scale spatial patterns in both risk maps. A clear geographic trend is apparent, ranging from the North where communities tend to only have a small group of individuals who engage in high-risk behavior, to the South where a high proportion of individuals in a community engage in high-risk behavior. Urban communities throughout the country and fishing communities around Lake Malawi have the highest percentage of high-risk individuals. However, most individuals who belong to the HRG live in rural areas, ~ 80% of high-risk women and ~ 85% of high-risk men. This is due to the fact that most of Malawi’s population lives in rural areas.

The SER models were a better fit to the MDHS data than the corresponding non-spatial models (Table 1). The spatial models show for both women and men that, at the level of the district, there is a significant geospatial correlation between HIV prevalence and the size of the HRG: as the size of the HRG increases, the severity of the epidemic increases. Seventy-three percent of the geographic variation in the severity of the epidemic in women can be explained by geographic variation in the size of their HRG. Slightly less, 67%, of the geographic variation in the severity of the epidemic in men can be explained by geographic variation in the size of their HRG.

**Table 1:** Results from the spatial and non-spatial district-level regression models: ordinary least squares regression (OLSR) and spatial error regression (SER). The SER model includes a spatially auto-correlated error term which accounts for the fact that variables that are geographically close are more likely to be similar. The size of the HRG in each district, for women, is defined as the proportion of women (15–49 years old) in the district who have had three or more lifetime sex partners (LSPs). The size of the HRG in each district, for men, is defined as the proportion of men (15–49 years old) in the district who have had four or more LSPs

	HIV prevalence, women		HIV prevalence, men	
	OLSR	SER	OLSR	SER
Size of the high-risk group (HRG)	0.53***	0.33***	0.30**	0.19***
Constant	0.04	0.06	–0.02	0.02
Lambda	NA	0.63***	NA	0.73***
AIC	–105	–112	–104	–120
*R*-squared	0.60	0.73	0.28	0.67

Lambda represents the level of auto-correlation in the error term

Asterisks denote the significance level according to the following *P*-values: ***P < 0.001, **P < 0.01

*AIC* Akaike information criterion

**Fig. 4:**
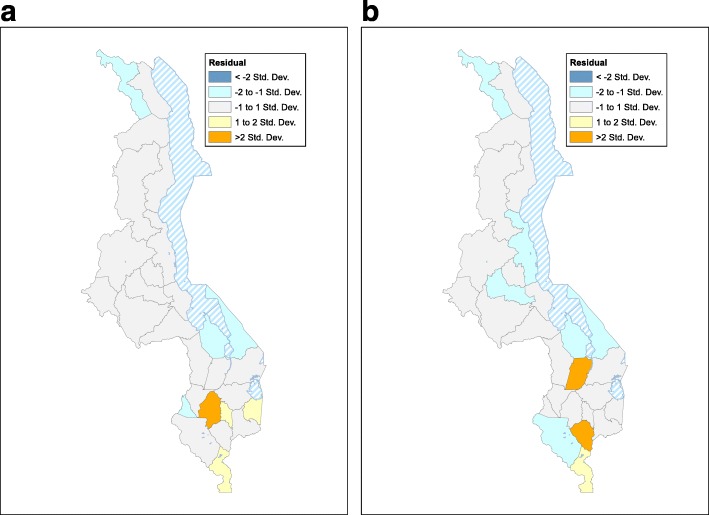
**a** Residuals from the spatial error regression model for women. The 20 districts where the residual is less than one standard deviation are shown in *gray*. The remaining colors show the degree to which the model under/overestimates prevalence in terms of the number of standard deviations. **b** Residuals from the spatial error regression model for men. The 19 districts where the residual is less than one standard deviation are shown in *gray*. The remaining colors show the degree to which the model under/overestimates prevalence in terms of the number of standard deviations

The SER models show that HIV prevalence in women can be expected to exceed the national average (~ 13%) in districts where ~ 20% or more of women are in the HRG. Similarly, the models show that prevalence in men can be expected to exceed the national average (~ 8%) in districts where ~ 30% or more of men are in the HRG. The residual maps show the error of the spatial models in estimating prevalence (Fig. 4a for women, Fig. 4b for men). In the majority of the 27 districts (20 for women, 19 for men) the models fit extremely well; the residual is less than one standard deviation (SD), shown by the gray areas. The models underestimate prevalence, residual > 2 SD, for women in one district and for men in two districts; these districts are shown in orange.

## Discussion

Our study shows that there is substantial geographic variation in the size of the HRG of both women and men throughout Malawi, and that this variation can be observed as large-scale geospatial patterns. We have found similar large-scale geospatial patterns for HIV prevalence. Most importantly, we have identified a statistically significant geospatial relationship between the size of the HRG and HIV prevalence. The geostatistical model that we have developed shows that the larger the size of the HRG, the more severe the epidemic. The quantitative results from the model demonstrate the importance of this relationship: they show that a substantial proportion (73% for women, 67% for men) of the geographic variation in HIV prevalence can be explained by geographic variation in the size of the HRG. Taken together, our results provide a mechanistic explanation for the large-scale countrywide variation in the severity of the HIV epidemic in Malawi.

Notably, the objective of our analysis is not — as others have done in previous studies [6, 11–13] — to construct a model to predict prevalence. Instead, our objective is to use geostatistical modeling to test a specific hypothesis. Consequently, we have designed a parsimonious geostatistical model that includes only one variable. To develop a predictive model, it would be necessary to include additional biological and behavioral variables that geographically covary with prevalence. These could be biological and/or behavioral cofactors, e.g., the presence of herpes simplex virus (HSV-2) or other sexually transmitted diseases, condom usage, and mobility patterns [20–25]. Notably, the level of medical circumcision (which reduces the risk of men acquiring HIV) is extremely low in Malawi; only 2.2% of men 15–49 years old are medically circumcised. Therefore, circumcision should not be included (as an explanatory factor) in any model for predicting prevalence in Malawi.

Our results provide new insights into the spatial diffusion of the HIV epidemic in Malawi and highlight the importance of mobility networks. Notably, we found that the majority of HIV-infected individuals have not engaged in high-risk behaviors. Many HIV-infected women have only had one or two lifetime sex partners. Our maps reveal that HIV-infected individuals are dispersed throughout Malawi and live in all types of demographic communities: urban, semi-urban, and rural. All of these communities, at some point, must have “imported” HIV. In Malawi, as in many other countries in sub-Saharan Africa, populations are highly mobile, and travelers, in comparison with non-travelers, have been shown to have an increased risk of HIV infection [21, 26, 27]. HIV-infected travelers are likely to have been (and continue to be) extremely important in linking high-prevalence urban centers and/or the fishing villages along Lake Malawi with low-prevalence rural communities. Phylogenetic analysis could be used to differentiate between localized and imported strains [28–30] and determine, for any specific community, where transmission is occurring.

The data we have used are the most appropriate for testing our hypothesis, as treatment coverage in 2010 in Malawi was fairly low; coverage is now fairly high at ~ 50% [31–33]. Increasing coverage, by increasing survival, increases prevalence; consequently, more recent data may obscure the relationship between prevalence and the size of the HRG. As with all studies, ours has limitations. We have found a geostatistical association between the size of the HRG and prevalence, but this does not necessitate causation; we do not know where transmission occurred. Sexual behavior data are not always accurate; women may under-report, and men over-report, their number of partners [34]. This can be problematic if an analysis necessitates classifying individuals into one of many behavioral risk groups. However, we use only two groups, and we define the HRG based on a relatively low number of lifetime sex partners. Consequently, we believe that it is unlikely that individuals were misclassified. An additional potential limitation is that female sex workers, who have very high numbers of partners, may not have participated in the MDHS. However, sex workers in Malawi only constitute 1% of the female population [35]. Accordingly, non-participation by sex workers is unlikely to have biased our results. It would have had little effect on the size of the HRG, or prevalence, in any specific location.

## Conclusions

Our results have significant implications for the design of HIV epidemic control strategies in Malawi and potentially in other countries in sub-Saharan Africa. We have found that the epidemic is the most severe in the major urban centers in Malawi and that these areas have the highest concentration of individuals who are in the HRG. These results highlight the necessity of focusing prevention efforts on urban areas, which the Joint United Nations Programme on HIV and AIDS (UNAIDS) has begun to address in its global “cities” campaign [36]. However, we have shown that most HIV-infected individuals, and the majority of women and men who are in the HRGs, live in rural areas. These results demonstrate that the majority of resources for treatment and interventions will need to be used in rural areas, where the burden of disease is greatest. Due to low population density and settlement dispersion patterns, it will be extremely challenging to design cost-effective HIV control strategies for Malawi.

## Abbreviations

ESP: Epidemic surface prevalence
HRG: High-risk group
MDHS: Malawi Demographic and Health Survey
SER: Spatial error regression
